# Admission Patterns and Patient Outcomes in Intensive Care Delivery at a Rural Tertiary Hospital in Southern Nigeria: A Five-Year Retrospective Review

**DOI:** 10.7759/cureus.73251

**Published:** 2024-11-07

**Authors:** Ehi-Iyoha Iyoha, Harry Okwilagwe, Kenneth U Okonmah, Joseph Irabor, Oluwatunmise Olowo-Samuel, Segun V Aiyenuberun

**Affiliations:** 1 College of Medicine, Ambrose Alli University, Ekpoma, NGA; 2 Research Unit, Internal Medicine Interest Group of Nigeria, Lagos, NGA

**Keywords:** admission patterns, africa, icu mortality, intensive care, nigeria, outcomes

## Abstract

Background

Intensive care units (ICUs) play a vital role in managing critically ill patients, yet data on admission patterns and outcomes in rural healthcare settings, particularly in Nigeria, remain limited. Understanding these patterns is essential for improving patient care, especially in resource-limited, rural settings. This study aimed to investigate the admission and outcome patterns of patients admitted to the ICU at Irrua Specialist Teaching Hospital (ISTH), located in a rural part of Edo State, Nigeria.

Method

This was a retrospective study conducted at ISTH. Data on biodata, primary admission diagnosis, duration of admission, and patient outcomes were collected from the ICU register for all patients admitted to the ICU over a five-year period, from January 1, 2019, to December 31, 2023, and analyzed.

Results

During the five-year study period, there were 581 ICU admissions, of which 575 (99%) were included in the study after excluding six (1%) due to incomplete data. Among the patients, 297 (51.7%) were female. The young and middle-aged group (20-59 years) accounted for 60% (365) of admissions. Surgical cases comprised the majority of admissions at 57.9% (333), followed by medical cases at 25.9% (149) and obstetric/gynecologic cases at 16.2% (93). The most common sources of admission were general surgery (211, 36.7%), obstetrics (75, 13%), and neurosurgery (43, 7.5%), with the most frequent diagnoses being generalized peritonitis secondary to perforated viscus (102, 17.7%), bowel obstruction (36, 6.3%), preeclampsia/eclampsia (28,4.9%), traumatic brain injury (TBI) (27, 4.7%), and stroke (26, 4.5%). The overall mortality rate was 49.2% and was significantly associated with age, duration of admission, type of case, and subspecialty (p < 0.05).

Conclusion

The study highlights admission and outcome patterns that reflect the challenges faced in rural ICU settings. The high mortality rate highlights the need for improved critical care resources and targeted interventions to improve outcomes of critically ill patients in similar resource-limited settings.

## Introduction

Intensive care, also known as critical care, is a form of highly specialized medical care focused on the management of critically ill patients who have, are at risk of, or are recovering from life-threatening conditions [1]. This is typically provided in a separate, dedicated section of the hospital called the intensive care unit (ICU) or critical care unit (CCU) [2]. The primary goal of intensive care is to maximize the chances of survival and facilitate the recovery of critically ill patients [1].

Despite the importance of ICUs, the high costs of the necessary personnel, infrastructure, and supplies have hindered their development in low-income countries [3]. This has created a disparity, with low-income countries having fewer ICU beds per capita compared to high-income nations [4]. This lack of ICU resources is even more pronounced in rural areas, where the majority of the population in low-income countries reside, as the limited ICU capacity available is usually concentrated in the urban centers of these countries. This issue is compounded by the high and rising burden of disease, especially non-communicable diseases, in these low-income settings, leading to increased demand for intensive care [5].

This disparity in ICU availability has led to poorer outcomes in developing countries, with studies showing substantially higher mortality rates in African countries compared to more developed nations [6-14]. For example, ICU mortality rates in Malawi, Uganda, and Ethiopia stand at 60.9%, 40.1%, and 38.7%, respectively [6-8], far exceeding rates of 5.4%, 7%, and 11.3% observed in Australia, Singapore, and the United States, respectively [9-11]. In Nigeria, ICU mortality rates are similarly high, like the rest of the continent, with studies in Jos, Enugu, and Calabar reporting rates of 48.2%, 34.6%, and 32.9%, respectively [12-14]. These differences in outcomes are likely influenced by various factors, including ICU performance [3].

This study, therefore, was carried out to determine the sociodemographic profile of patients admitted to the ICU of Irrua Specialist Teaching Hospital (ISTH), a rural tertiary hospital in southern Nigeria, along with the outcomes of these admissions, primarily focusing on mortality and the factors influencing it. It also seeks to identify any unique patterns or challenges associated with intensive care delivery in rural facilities, as most prior research has focused on urban hospitals, which may not reflect the realities of rural healthcare. The findings from this study could inform strategies to improve ICU outcomes and guide the allocation of scarce resources in low-income countries, particularly in underserved rural areas.

## Materials and methods

Study design

This study was a five-year retrospective study of all patients admitted to the ICU of Irrua Specialist Teaching Hospital (ISTH), Edo State, Nigeria, from January 1, 2019, to December 31, 2023.

Study area

This study was conducted at the Irrua Specialist Teaching Hospital (ISTH), a 440-bed capacity tertiary healthcare institution situated in Edo State, in the South-South geopolitical zone of Nigeria. The hospital's location in Irrua, the administrative capital of Esan Central Local Government Area, positions it within a largely agrarian rural community.

The hospital's ICU has four beds equipped with mechanical ventilators, defibrillators, suction machines, patient monitors, and infusion pumps. The ICU is under the care of the hospital's Department of Anesthesia and is staffed by several consultant anesthetists, anesthesia residents, intensive care nurses, and ICU technicians, with the patients being managed by both the anesthetists and the admitting surgeon or physician.

Inclusion and exclusion criteria

All patients admitted to the ICU of ISTH during the study period, with complete medical records, were included in the study. Only those with incomplete or missing medical records were excluded.

Data collection

Data was extracted from the hospital's ICU admission and discharge registers and recorded on a proforma sheet designed for the study. Data extracted from the registers include age, sex, diagnosis, source of admission (subspecialty), duration of admission, and clinical outcome of all admissions from January 1, 2019, to December 31, 2023. Possible clinical outcomes include transfer to the wards, discharge against medical advice (DAMA), referral to another facility, discharge home, and death.

Data analysis

Data analysis was done using Microsoft Excel 2021 (Microsoft Inc., Redmond, WA). Descriptive statistics were calculated for the variables and expressed as frequencies and percentages in tables and graphs. The chi-square test was employed to assess the association between variables and mortality, with the significance level set at p < 0.05.

Ethical consideration

Ethical approval was granted for this study by the ISTH Health Research Ethics Committee, with protocol number ISTH/HREC/20243105/598.

## Results

A total of 581 patients were admitted to the ICU during the five-year study period; however, six patients were excluded due to incomplete data, resulting in 575 (99%) patients being included in the study. Among these 575 patients, 278 (48.3%) were male and 297 (51.7%) were female, giving a male-to-female ratio of 1:1.07. Age ranged from eight days to 96 years, with a mean (±standard deviation (SD)) age of 42.9 (±22.0) years. When categorized into age groups, young and middle-aged patients (aged 20-59 years) represented 60% (345) of total admissions, while the elderly population (aged 60-99 years) accounted for 25% (144) (Table 1).

**Table 1:** Sociodemographic profile of patients admitted to the ICU (N = 575) ICU: intensive care unit, SD: standard deviation

Variable	Mean ± SD/frequency (%)
Age (years)	42.9 ± 22.0
0-9	52 (9.0)
10-19	34 (5.9)
20-29	72 (12.5)
30-39	112 (19.5)
40-49	81 (14.1)
50-59	80 (13.9)
60-69	69 (12.0)
70-79	50 (8.7)
80-89	18 (3.1)
90-99	7 (1.2)
Sex	
Male	278 (48.3)
Female	297 (51.7)
Year of admission	
2019	139 (24.2)
2020	106 (18.4)
2021	94 (16.3)
2022	134 (23.3)
2023	102 (17.7)
Duration of admission (days)	4.13 ± 5.3
≤1 day	229 (39.8)
2 days	89 (15.5)
3-7 days	184 (32.0)
8-14 days	44 (7.6)
>14 days	29 (5.0)

Regarding the duration of admission, 318 (55.3%) patients had an admission length of two days or less. The duration for all patients ranged from 30 minutes to 32 days, with a mean (±SD) duration of 4.13 (±5.3) days (Table 1).

Admissions were further classified by type: surgical, medical, or obstetric/gynecologic. Of the total admissions, 331 (57.9%) cases were classified as surgical, 149 (25.9%) as medical, and 93 (16.2%) as obstetric/gynecologic. The subspecialties with the highest admissions were general surgery (211, 36.7%), obstetrics (73, 12.7%), and neurosurgery (43, 7.5%) (Table 2).

**Table 2:** Types of cases and subspecialties admitted to the ICU (N = 575) *Including meningitis **Including pneumonia ***Poisoning and drug overdose, anaphylactic shock, electrolyte disorders, dermatologic emergencies ICU: intensive care unit

Variable	Frequency (%)	Mortality (%)
Types of cases		
Surgical	333 (57.9)	164 (49.2)
Medical	149 (25.9)	90 (60.4)
Obstetric and gynecologic	93 (16.2)	29 (31.2)
Subspecialties		
Surgical		
General surgery	211 (36.7)	106 (50.2)
Neurosurgery	43 (7.5)	31 (72.1)
Orthopedics	18 (3.1)	3 (16.7)
Plastic surgery and burns	17 (2.9)	8 (47.0)
Ear, nose, and throat/head and neck surgery	15 (2.6)	5 (33.3)
Urology	11 (1.9)	5 (45.4)
Pediatric surgery	8 (1.4)	5 (62.5)
Cardiothoracic surgery	7 (1.2)	1 (14.3)
Anesthesia	3 (0.5)	0 (0.0)
Medical		
Cardiology	37 (6.4)	21 (56.7)
Neurology*	33 (5.7)	21 (63.6)
Infectious diseases and sepsis	29 (5.0)	24 (82.7)
Pulmonology**	13 (2.3)	6 (46.1)
Hematology and hemato-oncology	8 (1.4)	5 (62.5)
Gastroenterology	5 (0.9)	3 (60.0)
Nephrology	4 (0.7)	2 (50.0)
Endocrinology	3 (0.5)	3 (100.0)
Others***	13 (2.3)	5 (38.5)
Obstetric and gynecologic		
Obstetrics	75 (13.0)	25 (33.3)
Gynecology	18 (3.1)	4 (22.2)

The six most common primary admission diagnoses were generalized peritonitis secondary to perforated viscus (102, 17.7%), bowel obstruction (36, 6.3%), preeclampsia/eclampsia (28, 4.9%), traumatic brain injury (TBI) (27, 4.7%), stroke (26, 4.5%), and sepsis (24, 4.2%) (Table 3).

**Table 3:** Most frequent primary admission diagnoses

Primary diagnosis	Frequency (%)	Mortality (%)
Generalized peritonitis secondary to perforated viscus	102 (17.7)	68 (66.7)
Bowel obstruction	40 (6.9)	17 (42.5)
Traumatic brain injury	27 (4.7)	20 (74.1)
Preeclampsia/eclampsia	28 (4.9)	11 (39.3)
Stroke	26 (4.5)	17 (65.4)
Sepsis	24 (4.2)	19 (79.2)
Abdominal trauma	23 (4.0)	19 (82.6)
Pulmonary embolism	17 (2.9)	10 (58.8)
Musculoskeletal trauma	16 (2.8)	2 (12.5)
Gastrointestinal malignancy	14 (2.4)	7 (50.0)
Postpartum hemorrhage	13 (2.3)	4 (30.8)
Heart failure	11 (1.9)	4 (36.4)
Upper airway obstruction	11 (1.9)	4 (36.4)
Burns	9 (1.6)	8 (88.9)
Maternal sepsis	9 (1.6)	2 (22.2)
Gynecologic malignancy	9 (1.6)	3 (33.3)
Thyroid disease	9 (1.6)	0 (0.0)
Pneumonia	7 (1.2)	2 (28.6)
Intra-abdominal abscess	7 (1.2)	3 (42.8)
Poisoning/drug overdose	5 (0.9)	3 (60.0)
Facial trauma	5 (0.9)	0 (0.0)
Sickle cell anemia and complications	5 (0.9)	2 (40.0)
Thoracic trauma	5 (0.9)	1 (20.0)
Breast disease	5 (0.9)	0 (0.0)
Placental abruption	4 (0.7)	2 (50.0)
Acute coronary syndrome	4 (0.7)	2 (50.0)
Acute respiratory distress syndrome	4 (0.7)	2 (50.0)
Status epilepticus/seizure disorder	4 (0.7)	2 (50.0)
Uterine rupture	4 (0.7)	1 (25.0)
Intracranial hemorrhage	4 (0.7)	2 (50.0)
Hypertensive emergency	3 (0.5)	1 (33.3)
Tetanus	3 (0.5)	3 (100.0)
Anaphylactic shock	3 (0.5)	1 (33.3)
Liver disease	3 (0.5)	3 (100.0)
Anesthetic complications	3 (0.5)	0 (0.0)

Among all admitted cases, there were 283 deaths, resulting in an overall mortality rate of 49.2%. A total of 265 (46.1%) were transferred to wards for continued care, while 14 (2.4%) were referred to other facilities for further management (Table 4).

**Table 4:** Outcomes of ICU admissions (N = 575) ICU: intensive care unit, DAMA: discharged against medical advice

Outcome of admission	Frequency (%)
Dead	283 (49.2)
Transferred to ward	265 (46.1)
Referred	14 (2.4)
Discharged home	8 (1.4)
DAMA	5 (0.9)

To evaluate the relationship between various factors and mortality, a chi-square analysis was performed. The results indicated that age, duration of admission, type of case, and subspecialty were significantly associated with mortality (p < 0.05) (Table 5).

**Table 5:** Chi-square analysis comparing mortality and sociodemographic variables (p < 0.05) (N = 575) *Statistically significant

Variable	Mortality (%)	x^2^	Df	P-value
Sex		0.6	1	0.420
Male	132 (47.5)			
Female	151 (50.8)			
Age (years)		17.9	4	0.001*
0-19	43 (50.0)			
20-39	66 (36.9)			
40-59	97 (58.4)			
60-79	65 (54.6)			
80-99	12 (48.0)			
Duration of admission (days)		4.4	1	0.036*
<2 days	125 (54.6)			
≥2 days	158 (45.7)			
Type of case		19.6	2	<0.001*
Surgical	164 (49.2)			
Medical	90 (60.4)			
Obstetric and gynecologic	29 (31.2)			
Subspecialty		30.6	5	<0.001*
General surgery	106 (50.2)			
Obstetrics	25 (33.3)			
Neurosurgery	31 (72.1)			
Cardiology	21 (56.7)			
Neurology	21 (63.6)			
Infectious diseases and sepsis	24 (82.7)			

## Discussion

The findings of this study offer valuable insights into the admission patterns and outcomes of patients in the ICU at our tertiary hospital over a five-year period. Our analysis included 575 patients, revealing a nearly balanced gender distribution with a slight predominance of females (51.7%). This contrasts with several studies conducted in Nigeria and Africa, where males are typically more prevalent [6-8,12-14]. The higher female admission rate at our center may be linked to the relatively high number of obstetric and gynecologic cases, which account for 16.2% of all ICU admissions. This trend is also observed in Abuja, where female admissions are higher, with a large proportion of obstetric cases at 24.5% [15]. In contrast, Ilori et al. report a predominance of male admissions, with obstetric cases at only 5.8% [14]. The larger number of obstetric admissions at our facility may be attributed to its rural location, which is characterized by limited antenatal care and few childbirth facilities, leading many women to rely on poorly skilled birth attendants, resulting in complications that necessitate referral to our tertiary center.

Our data indicate a mean monthly admission rate of approximately nine patients. In comparison, two other Nigerian studies conducted in Calabar and Abakaliki reported lower and higher figures of about seven and 14, respectively [14,16]. This variation can be attributed to differences in ICU bed capacity, with Calabar and Abakaliki having three and six beds, respectively, compared to our center's four beds. When adjusted for the number of beds, the admission rates in these centers align more closely with ours, yielding figures of nine and 10 admissions, respectively.

The age distribution in our study shows that a significant proportion of admissions were among young and middle-aged patients, accounting for 60% of total admissions. This trend is consistent with similar studies conducted in Nigeria and Sub-Saharan Africa [6,12,13,15]. Our mean age was 42.9 years, comparable to other Nigerian studies [13,14,17] but lower than those reported in high-income countries such as Australia (61.9 years) and the United States (57.6 years) [9,11], which can be attributed to the higher life expectancy in those regions.

The duration of ICU stays varied significantly, ranging from 30 minutes to 32 days, with more than half of the patients admitted for two days or less. While the high percentage of patients with short admissions may suggest effective triage and management strategies, allowing for rapid intervention and transfer to appropriate care settings, it also raises questions about the thresholds for ICU admission and the criteria used to determine the necessity of intensive care. This is particularly relevant given the absence of a high dependency unit (HDU) in our hospital, which necessitates that all patients requiring anything more than basic care be admitted to the ICU, regardless of whether their conditions truly warrant such a level of care. It is essential to evaluate whether patients with shorter stays truly benefit from ICU-level care or if they could be managed more effectively in alternative and more cost-efficient settings.

Towey and Ojara characterized the typical ICU in Sub-Saharan Africa as predominantly surgical [18], a description that holds true for our findings, as surgical cases comprised the majority of admissions (57.9%). This underscores the ICU's critical role in managing postoperative complications and severe surgical conditions. It also supports the need for ICUs to be located near operating theaters, which serve as their primary source of patients, thereby reducing the risks associated with transporting critically ill patients [19,20]. Additionally, this proximity enhances patient safety, as anesthetists in the operating theater, who also oversee the ICU, are readily available to intervene when necessary [14]. While surgical cases constitute the majority in our study, the percentage is relatively low compared to similar studies [13,14,16]. This discrepancy may arise because those studies classify obstetric and gynecologic cases as surgical. If these two categories were combined in our study, our rates would align more closely with those reported in these studies.

**Figure 1:**
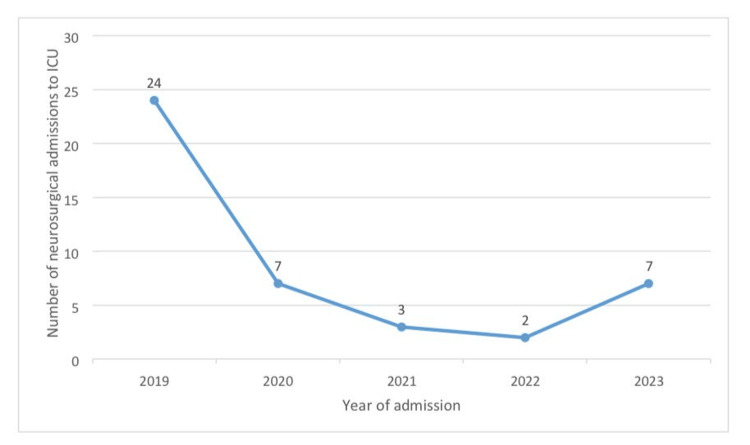
Trend of neurosurgical admissions to the ICU of ISTH over the study period ICU: intensive care unit, ISTH: Irrua Specialist Teaching Hospital

General surgery was identified as the leading subspecialty in terms of admissions. In contrast, studies conducted in Enugu, Nnewi, and Lagos report neurosurgery as having the highest admission rates [13,17,21]. In our study, neurosurgery ranked second, with over half of the admissions (24, 55.8%) occurring in a single year (2019) during the five-year study period, as shown in Figure 1. This decline can be attributed to the departure of the hospital's sole neurosurgeon in early 2020, which led to the referral of neurosurgical cases until a new neurosurgeon joined the hospital in late 2023, leading to a surge in neurosurgery admissions in that year, also shown in Figure 1. If neurosurgery activity had continued at the same rate as in 2019, it is possible that it would have become the highest subspecialty in terms of admissions. This observation aligns with the findings of Oji, who noted that admission patterns are influenced by the availability of specialists and the range of services offered by the institution [22].

Generalized peritonitis due to perforated viscus was the most frequent primary admission diagnosis, followed by bowel obstruction, traumatic brain injury (TBI), and preeclampsia/eclampsia. The high incidence of these cases in our study underscores the need for robust surgical and obstetric services to effectively manage these life-threatening conditions. While this contrasts with some studies where TBI was the leading diagnosis [13,17,21], it is consistent with findings from other studies that identified bowel perforation and obstruction as the most common cases [14,23].

The overall mortality rate of 49.2% is concerning. Although this rate is comparable to those reported in Nigerian and African studies, generally ranging from 32.9% to 60.9% [6-8,12-14], it is significantly higher than rates observed in high-income countries [9-11]. Mato et al. notably reported a lower mortality rate of 24.3%, but this was attributed to about 41.5% of ICU admissions being non-justifiable as they were due to a lack of bed space in the general wards [24]. Furthermore, medical cases demonstrated higher mortality rates compared to surgical and obstetric and gynecologic cases, with stroke and sepsis being the most frequent medical diagnoses, both associated with very high case fatality rates of 65.4% and 79.2%, respectively; similar patterns have also been noted in other studies [14-16,21].

Our study revealed that age, duration of admission, type of case, and subspecialty were significantly associated with mortality, as indicated by the chi-square analysis. This suggests that targeted interventions aimed at high-risk groups, particularly those in specific age brackets or those with prolonged ICU stays or specific disease conditions, may be beneficial in reducing mortality rates.

Limitations

This study has several limitations that should be acknowledged. As a single-center study, the findings may not be generalizable to other rural tertiary hospitals or different healthcare settings. The retrospective nature of the study meant relying on previously collected patient records, some of which were incomplete. Additionally, the study did not take into account several factors, including socioeconomic status, interventions such as mechanical ventilation, inotropic support, blood transfusions, comorbid conditions, and disease severity scoring, all of which could significantly influence patient outcomes. Despite these limitations, this study provides valuable insights into ICU admission patterns and outcomes, which could enhance the targeting of interventions tailored to the specific challenges encountered in rural healthcare settings while also serving as a useful baseline for future research in this field.

We recommend that future research endeavors aim to address these limitations by conducting multicenter studies for more holistic results, employing prospective data collection methods to address issues of incomplete records, and including more variables such as socioeconomic status, interventions, and disease severity scoring to better understand their effects on patient outcomes.

## Conclusions

In conclusion, this study provides important insights into the admission patterns and outcomes of ICU patients at our rural tertiary hospital. The findings highlight the predominance of surgical cases, as well as the significant mortality rates associated with specific age groups and medical conditions. Given the challenges posed by limited healthcare resources in rural areas, targeted interventions aimed at high-risk groups are essential to improve patient outcomes. By addressing the unique needs of the rural population and focusing on enhancing access to care, we can work toward reducing mortality rates and improving the overall quality of healthcare in these underserved regions.
